# Adipokine profiles and genetic variants of leptin receptor, adiponectin, and ghrelin pathways in obesity: prospective 12-month outcomes after bariatric interventions

**DOI:** 10.3389/fendo.2026.1841033

**Published:** 2026-05-20

**Authors:** Andriy Prodan, Ihor Dzyubanovsky, Iryna Halabitska, Pavlo Petakh, Iryna Kamyshna, Oleksandr Kamyshnyi

**Affiliations:** 1 Department of Surgery of Faculty of Postgraduate Education, I. Horbachevsky Ternopil National Medical University, Ternopil, Ukraine; 2 Department of Therapy and Family Medicine, I. Horbachevsky Ternopil National Medical University, Ternopil, Ukraine; 3 Kyiv School of Economics, Kyiv, Ukraine; 4 Department of Biochemistry and Pharmacology, Uzhhorod National University, Uzhhorod, Ukraine; 5 Department of Medical Rehabilitation, I. Horbachevsky Ternopil National Medical University, Ternopil, Ukraine; 6 Department of Microbiology, Virology, and Immunology, I. Horbachevsky Ternopil National Medical University, Ternopil, Ukraine

**Keywords:** adipokines, bariatric surgery, body mass index, genetic polymorphisms, metabolic disorders, obesity

## Abstract

**Introduction:**

Adipokines and genetic factors play an important role in the development of obesity and its metabolic complications. The study aimed to assess the relationships among genetic polymorphisms, adipokine levels, and clinical and metabolic indicators in patients with varying degrees of obesity, and to analyze changes in anthropometric and metabolic parameters after bariatric interventions.

**Methods:**

A prospective observational study included 76 adult patients with obesity, divided by body mass index. Anthropometric indicators, glycated hemoglobin levels, and adipokine concentrations (leptin, adiponectin, resistin, and total ghrelin) were determined. Genotyping of polymorphisms (rs1137101, rs1137100, rs7799039, rs266729, rs17300539, rs1805094, rs696217) was performed using standard molecular genetic methods. Patients underwent bariatric surgery (endovascular embolization, gastroplication, or sleeve gastrectomy) with a 12-month follow-up.

**Results:**

With increasing obesity, resistin and leptin levels increase, while adiponectin and ghrelin levels decrease (p < 0.05). The rs1137101 and rs696217 polymorphisms are associated with the degree of obesity: the A allele of rs1137101 is less common in patients with stage III. At the same time, the GG genotype is more prevalent in critically ill patients (p < 0.01). Bariatric interventions effectively reduce BMI and body weight, improve glycemic control, and normalize the hormonal profile (p < 0.001). The T allele of rs696217 and G of rs1137101 affect weight dynamics and hormonal changes after surgery.

**Conclusions:**

The hormonal profile and genetic variants of rs1137101 and rs696217 affect the severity of obesity and the effectiveness of bariatric treatment. Genetic testing may help predict outcomes and personalize therapy.

## Highlights

- Hormonal changes: Resistin and leptin levels increase, while adiponectin and ghrelin levels decrease with increasing obesity.
- Genetic predisposition: The rs1137101 (LEPR) and rs696217 (GHRL) polymorphisms are associated with severe obesity; the A allele of rs1137101 is less frequent, and the GG genotype is more common in critically ill patients.
- Effectiveness of bariatric interventions: Surgery significantly reduces BMI, improves glycemic control, and normalizes hormonal profiles.
- Influence of genetics on outcome: The T allele of rs696217 is associated with greater weight loss and hormonal changes; the G allele of rs1137101 affects adiponectin, resistin, and ghrelin levels.

## 1 Introduction

Obesity is associated with an increased risk of developing metabolic disorders, including insulin resistance, type 2 diabetes, and cardiovascular disease (1–4). The development of this condition is due to complex interactions among genetic, hormonal, and behavioral factors (5–7).

Adipose tissue plays an important role in regulating metabolic processes. It functions as an active endocrine organ and synthesizes various biologically active molecules known as adipokines (8, 9), including leptin, adiponectin, and resistin. These hormones are involved in the regulation of appetite, energy balance, inflammation, and carbohydrate metabolism (10–12). In addition to adipokines, other hormones involved in energy homeostasis, such as ghrelin, also contribute to metabolic regulation. Although primarily produced in the gastrointestinal tract, ghrelin plays a key role in appetite control and energy balance and interacts with adipokine-mediated pathways (13–15). Alterations in the levels and signaling of these hormones are commonly observed in obesity and may contribute to the development and progression of metabolic complications (16–18).

Given the growing prevalence of obesity and its metabolic complications, increasing attention has been paid to genetic polymorphisms involved in adipokine signaling and metabolic regulation (19, 20). Variants rs1137101, rs1805094 and rs1137100 (LEPR), rs7799039 (LEP), rs266729 and rs17300539 (ADIPOQ), and rs696217 (GHRL) are associated with changes in the signaling activity of leptin, adiponectin, ghrelin, which may affect the susceptibility to obesity and the development of metabolic disorders (21–26). Genetics influences the susceptibility to obesity, its severity, and metabolic manifestations (27–29). The study of such genetic variants helps better understand the mechanisms of disease development and the individual characteristics of its course (30–32).

For example, the LEPR rs1137101 (Q223R) polymorphism may impair leptin receptor signaling and contribute to leptin resistance, while ADIPOQ variants influence adiponectin expression and insulin sensitivity (33, 34). The GHRL rs696217 polymorphism has been associated with altered ghrelin secretion and appetite regulation (35). These molecular mechanisms may underlie inter-individual variability in metabolic phenotypes and response to obesity treatment.

At the molecular level, obesity-related metabolic complications are mediated by complex genetic and epigenetic mechanisms involving adipokine signaling, inflammation, and energy homeostasis pathways (36, 37). Single-nucleotide polymorphisms (SNPs) in genes encoding adipokines and their receptors, such as LEPR, ADIPOQ, and GHRL, may alter receptor binding affinity, intracellular signaling (e.g., JAK/STAT, PI3K/Akt pathways), and hormone secretion (38, 39).

One of the most effective methods of treating severe obesity is bariatric surgery (40, 41). These interventions result in a significant reduction in body weight and improvements in metabolic parameters. In addition, after surgery, changes occur in the hormonal profile and adipokines levels, which may play an important role in improving the metabolic status of patients (42–44).

Despite the growing body of literature on adipokines and genetic determinants of obesity, there remains a limited understanding of how genetic polymorphisms interact with longitudinal changes in adipokine profiles following bariatric interventions. In particular, data on the combined predictive value of these biomarkers for metabolic outcomes after different types of bariatric procedures are scarce and often inconsistent. Therefore, a clearer integration of genetic and hormonal factors into surgical treatment is needed.

Unlike previous studies, this work integrates genetic polymorphisms with longitudinal adipokine dynamics and clinical outcomes following different bariatric procedures. Additionally, it evaluates the predictive role of genetic variants in modulating both metabolic response and weight-loss trajectories over a 12-month follow-up.

This study aimed to evaluate the relationships among genetic polymorphisms, adipokine levels, and clinical and metabolic parameters in patients with varying degrees of obesity. Changes in anthropometric and biochemical parameters after different types of bariatric interventions were also analyzed.

## 2 Materials and methods

### 2.1 Study design and population

The study had a prospective observational design and included adult patients with obesity. A total of 76 obese individuals were included in the analysis. Among them, 56 women (73.68%) and 20 men (26.32%) were included. The control group consisted of 48 non-obese individuals with body mass index within the normal range according to World Health Organization (WHO) criteria; 34 were women (70.83%), and 14 were men (29.17%). The groups were comparable by gender (p=0.653, χ²). The mean age of participants in the study group was 42.3 ± 10.5 years, while in the control group it was 40.8 ± 9.7 years, with no statistically significant difference between the groups (p=0.420). Control participants were recruited from individuals undergoing routine preventive medical examinations. They had no history of obesity, diabetes, or other significant metabolic disorders. Participants with obesity were stratified by severity according to the World Health Organization classification based on body mass index. Grade I obesity was detected in 5 patients, grade II in 28, and grade III in 43.

Inclusion criteria were age ≥18 years, the presence of clinically confirmed obesity, and signed informed consent. Patients with acute inflammatory diseases, severe decompensated chronic conditions, or incomplete clinical data were excluded from the analysis.

No formal sample size calculation was performed prior to the study; therefore, the sample can be considered a convenience sample based on available eligible participants.

The study included both cross-sectional comparisons at baseline and longitudinal follow-up assessments over a 12-month period.

### 2.2 Anthropometric and clinical assessment

Anthropometric parameters were determined according to a standardized protocol. Body weight and height were measured using calibrated equipment, and body mass index was calculated. Clinical examination included assessment of anthropometric parameters, carbohydrate metabolism parameters, and metabolic profile.

### 2.3 Biochemical and hormonal studies

Leptin, adiponectin, resistin, and ghrelin levels were determined by ELISA using Leptin ELISA kit (LDN Labor Diagnostics Nord GmbH & Co. KG, Germany), Human Ghrelin ELISA Kit, Human Adiponectin ELISA Kit, and Resistin Human ELISA Kit (Thermo Fisher Scientific, USA) on a Multiskan FC analyzer (Skanlt Software 4.1, wavelength 620 nm). Glycated hemoglobin (HbA1c) was measured by the enzymatic method on a Hitachi automated biochemical analyzer using Roche Diagnostics reagents and calibrators. Blood samples were collected in the morning after an overnight fast of at least 8–12 hours.

All assays were performed in duplicate, and mean values were used for analysis. Calibration curves were generated according to manufacturer instructions. Internal quality controls were included in each run. The detection limits and intra-/inter-assay coefficients of variation were within acceptable ranges as specified by the manufacturers.

### 2.4 Genotyping of the GHRL, LEP and LEPR genes polymorphism (rs696217, rs7799039, rs1137100, rs1137101, rs1805094)

Venous blood samples for genomic study were collected in tubes with ethyl-enediaminetetraacetic acid (EDTA). The ThermoScientific™ GeneJET™ Whole Blood Genomic DNAPurification Kit (Thermo Fisher Scientific, USA) wasused for genomic DNA extraction according to the manufacturer’s instructions. Pre-designed TaqMan™SNP Genotyping Assays, Human, (Thermo FisherScientific, USA) were used for next SNPs: K109R(rs1137100), Q223R (rs1137101), K656N (rs1805094),G2548A (rs7799039), C214A (rs696217). TaqMan™Universal Master Mix II, no UNG were used for DNA amplification using real-time polymerase chain reaction (PCR) technique of allele discrimination based on the magnitude of relative fluorescence units(RFU).

### 2.5 Bariatric surgery and follow-up

All included patients underwent bariatric interventions according to clinical indications. In particular, endovascular bariatric embolization (BE) was performed in 7 patients, gastric plication (GP) in 37 patients, and sleeve gastrectomy (SG) in 32 patients.

Surgical procedures were performed according to standardized clinical protocols. Patient selection for each procedure was based on BMI, comorbidities, and multidisciplinary evaluation. Postoperative management included nutritional counseling, lifestyle modification, and regular clinical monitoring. Adherence to follow-up was ensured through scheduled visits at 3, 6, and 12 months.

After surgery, patients were prospectively followed up with repeated anthropometric and laboratory measurements at specified time points during the follow-up period. The inclusion of different bariatric procedures reflects real-world clinical practice; however, this heterogeneity may influence metabolic and hormonal outcomes and should be considered when interpreting the results.

### 2.6 Statistical analysis

Statistical analysis was performed in Statistica 13.0, GraphPad Prism 9.0, and R (v4.3.0). Continuous variables are presented as M ± SD with 95% CI; categorical variables are presented as n (%). Normality was tested by the Shapiro–Wilk test. For comparisons of three or more groups, the Kruskal–Wallis test with Dunn’s *post hoc* test (Bonferroni correction) was used; for repeated measures, the Friedman test; for two independent groups, the Mann–Whitney U test. Categorical variables were analyzed using the Pearson χ² test; compliance of SNP genotypes with Hardy-Weinberg equilibrium was tested using the χ² test. Allele and genotype frequencies were calculated for each SNP, and associations with obesity were assessed in different genetic models. Correlations were determined using Pearson or Spearman coefficients, and the results were visualized in heat maps. All tests were two-sided; p < 0.05 was considered statistically significant.

### 2.7 Ethical aspects

The study was conducted in accordance with the ethical principles of the Declaration of Helsinki. The study protocol was approved by the Bioethics Commission of the I. Horbachevsky Ternopil National Medical University (protocol No. 12 dated November 4, 2020), and all participants provided written informed consent to participate in the study.

## 3 Results

### 3.1 Genetic, anthropometric, and metabolic parameters depending on the degree of obesity

In terms of hormonal parameters, resistin and leptin levels increased with obesity severity (p = 0.025 and p < 0.001, respectively), whereas adiponectin and ghrelin levels decreased (both p < 0.001). HbA1c showed a tendency to increase; however, the difference did not reach statistical significance (p = 0.064) (**Table 1****).**

**Table 1:** Levels of adipokines and HbA1c according to obesity severity.

Resistin, ng/ml	1st (n=5)	2nd (n=28)	3rd (n=43)	p
	7.76 ± 0.63	8.30 ± 0.75	9.00 ± 1.57	0.025
Adiponectin, µg/ml	7.70 ± 0.43	6.52 ± 0.67	5.83 ± 0.44	< 0.001* p_1st – 2nd_ < 0.001 p_1st – 3rd_ < 0.001 p_2nd – 3rd_ < 0.001
Ghrelin general, ng/ml	1157.64 ± 302.65	789.75 ± 208.47	654.68 ± 172.35	< 0.001* p_1st – 2nd_ < 0.001 p_1st – 3rd_ < 0.001 p_2nd – 3rd_ = 0.016
Leptin, ng/ml	18.28 ± 7.50	29.84 ± 20.28	55.90 ± 21.60	< 0.001* p_1st – 3rd_ < 0.001 p_2nd – 3rd_ < 0.001
HbA1c, %	5.54 ± 0.36	6.14 ± 0.53	6.12 ± 0.55	0.064

Data are mean ± s.d. Differences between groups were assessed using the Kruskal–Wallis test with Dunn’s *post hoc* test with Bonferroni correction for pairwise comparisons.

Regarding genetic analysis, SNP analysis revealed statistically significant differences for rs1137101, while rs696217 showed no consistent differences between groups. All studied SNPs were in Hardy–Weinberg equilibrium (p > 0.05).

**Figure 1:**
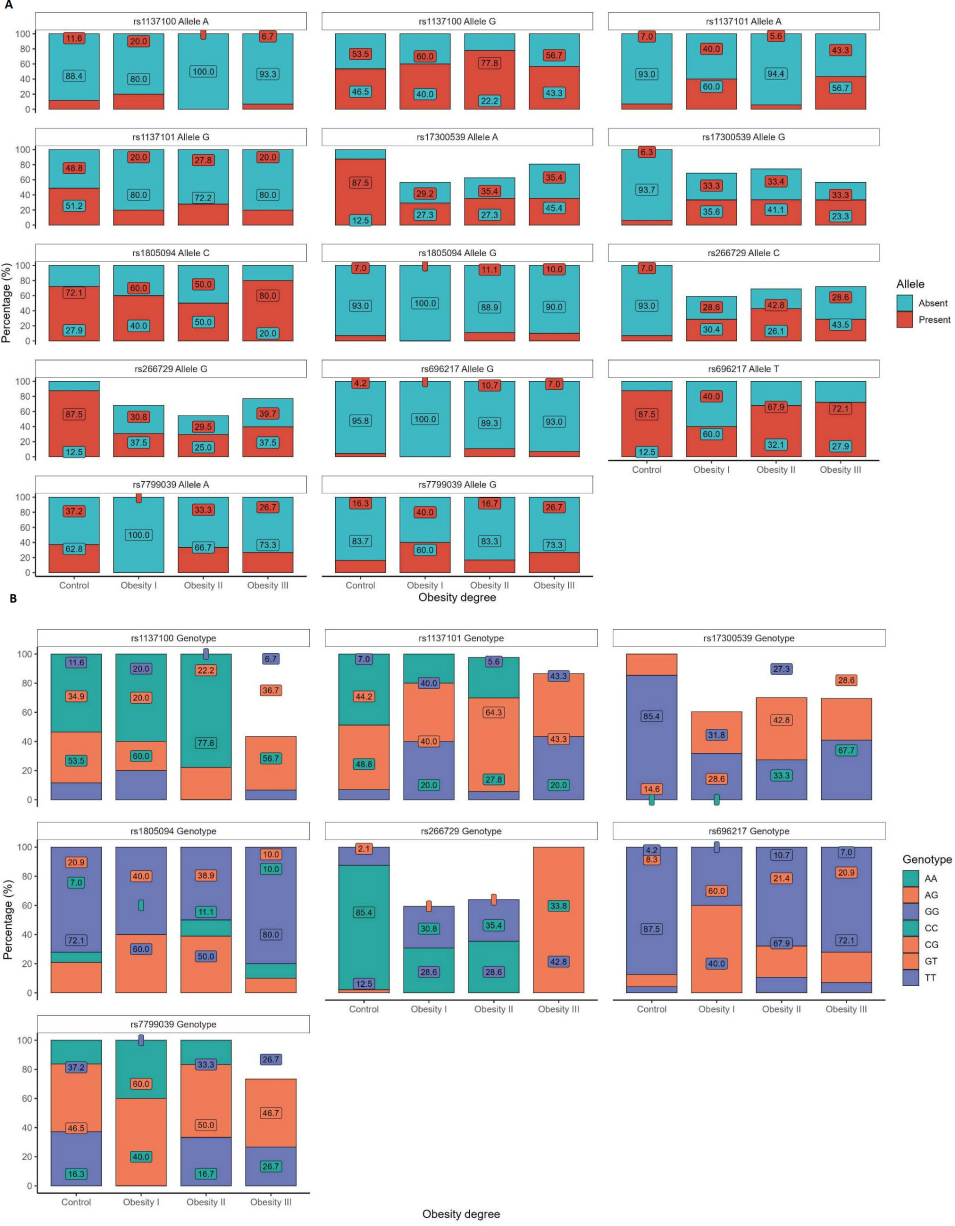
Allele and genotype distribution of obesity-related SNPs across obesity severity groups. **(A)** Allele frequencies of rs1805094, rs7799039, rs1137101, rs1137100, rs696217, rs266729, and rs17300539. **(B)** Genotype distribution of the same polymorphisms according to obesity degree.

The frequency of the A allele of rs1137101 was significantly lower in patients with grade III obesity compared to controls and patients with grade II obesity (p < 0.001). Correspondingly, the GG genotype was more prevalent in patients with grade III obesity, whereas the AA genotype predominated in the control group (p = 0.002). Both allele- and genotype-based analyses consistently demonstrated an association of rs1137101 with obesity severity (**Figure 1****).**

**Figure 2:**
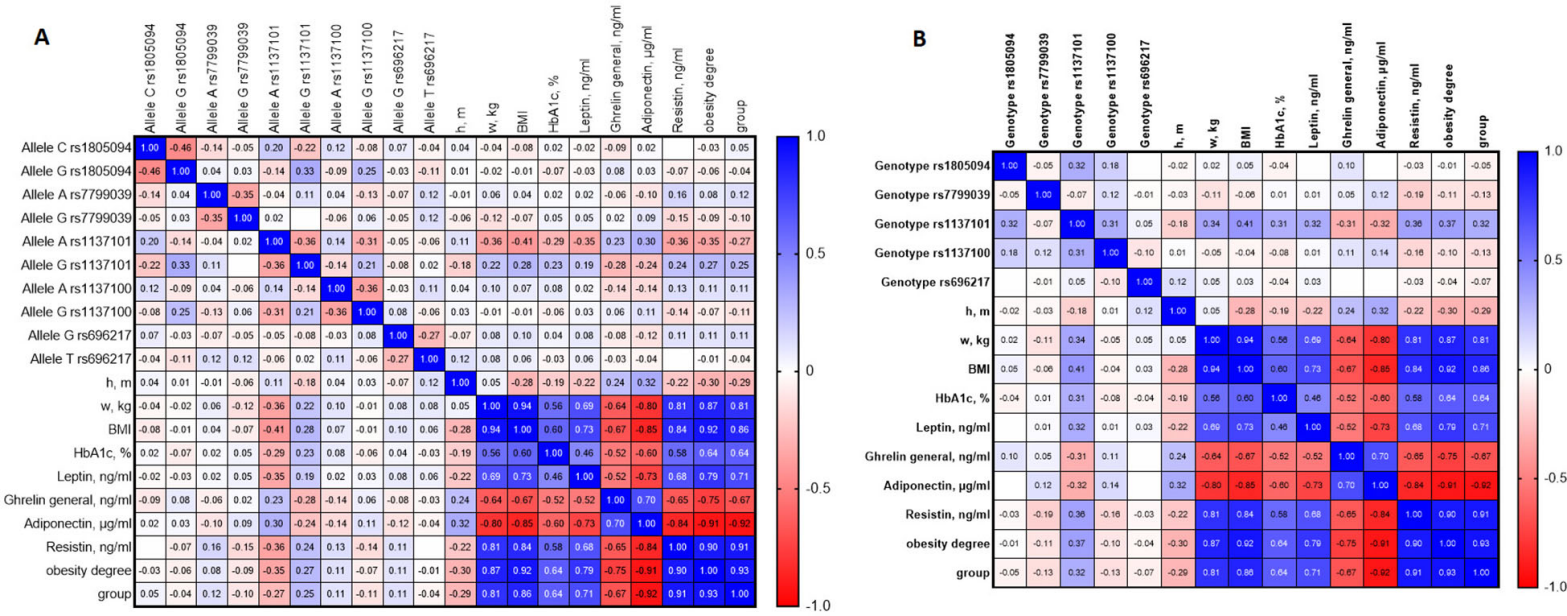
Correlation matrix of allelic variants, genotypes, and metabolic–hormonal traits associated with obesity. **(A)** Сorrelations calculated for individual alleles of the studied SNPs and clinical–metabolic parameters. **(B)** Сorrelations calculated for genotypes of the same SNPs and clinical–metabolic parameters.

Correlation analysis further demonstrated that rs1137101 was moderately associated with BMI (r = 0.41) and obesity grade (r = 0.37; p < 0.01). BMI strongly correlated with body weight, obesity grade, and clinical group (r = 0.81–0.94; p < 0.001). Leptin levels were positively correlated with BMI (r = 0.69–0.79; p < 0.001), whereas adiponectin and ghrelin showed strong negative correlations (r = −0.80 to −0.91 and r = −0.64 to −0.75, respectively; p < 0.001). HbA1c demonstrated a moderate positive correlation with BMI (r = 0.56–0.64; p < 0.001). Notably, some of the observed correlations were relatively strong and should be interpreted with caution, taking into account the sample size and potential confounding factors (**Figure 2****).**

### 3.2 Changes in anthropometric and metabolic parameters after bariatric surgery

**Figure 3:**
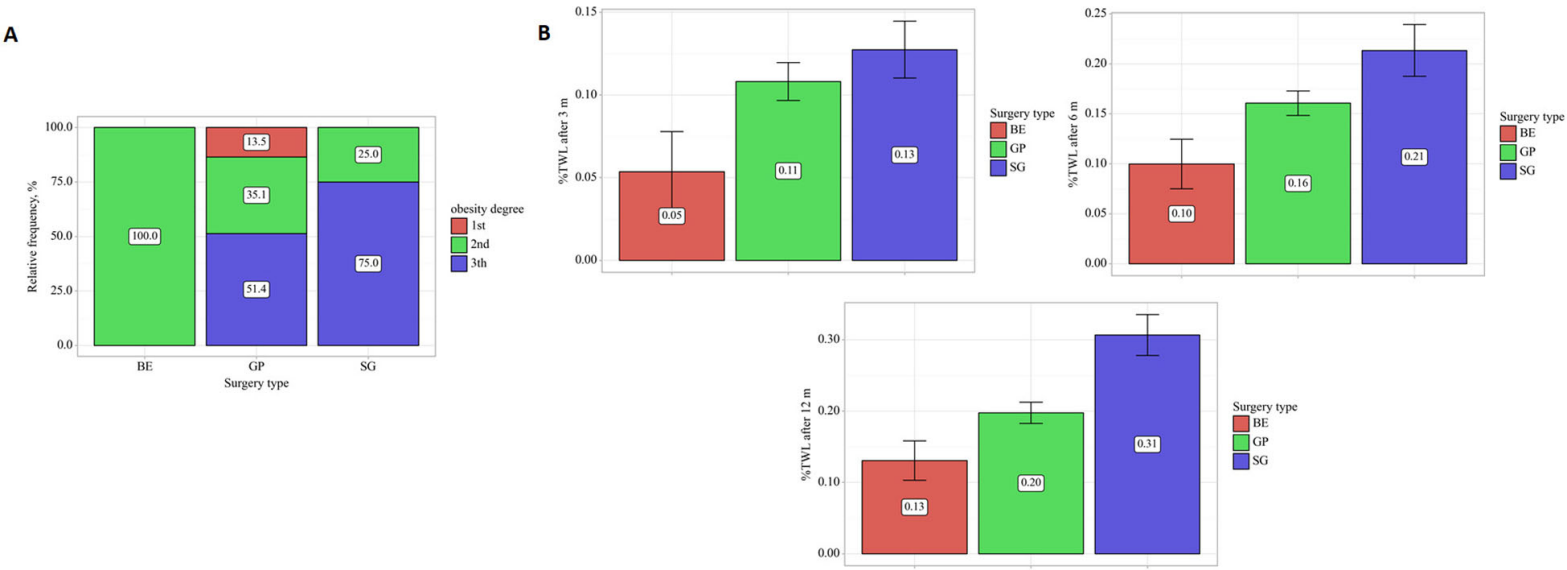
Distribution of obesity severity and changes in anthropometric outcomes across bariatric procedures. **(A)** Relative distribution of obesity severity grades among patients undergoing different bariatric procedures (BE, GP, SG). **(B)** Mean %TWL, percentage total weight loss at different follow-up time points according to surgery type, with error bars indicating variability.

The degree of obesity differed depending on the type of surgery (p < 0.001): in the BE group, all patients had obesity grade II, in the GP – all grades, and in the SG, obesity grade III predominated; a significant difference was observed between BE and GP (p = 0.020) (**Figure 3A**).

**Figure 4:**
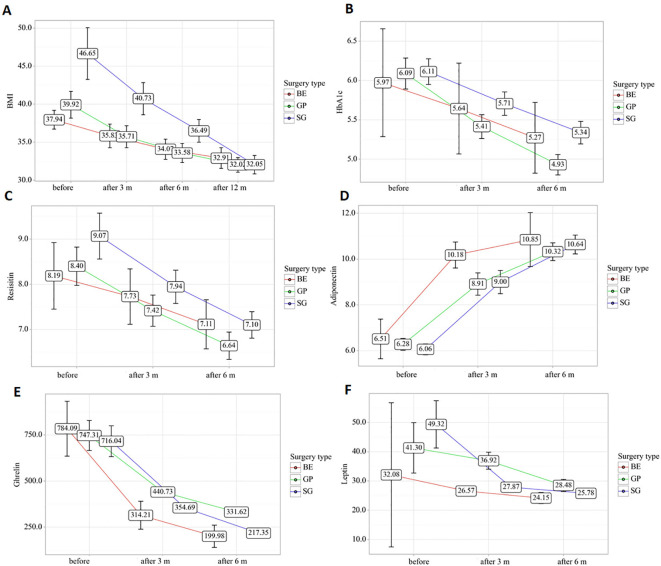
**(A)** Changes in body mass index (BMI) during 12 months of follow-up after bariatric embolization (BE), gastric plication (GP), and sleeve gastrectomy (SG). **(B)** Changes in glycated hemoglobin (HbA1c) levels over 6 months after surgery. **(C)** Dynamics of resistin concentrations during follow-up. **(D)** Changes in adiponectin levels after bariatric interventions. **(E)** Changes in total ghrelin concentrations over time. **(F)** Longitudinal changes in leptin levels after surgery. Data are presented as mean ± SD. Error bars indicate standard deviations. Measurements were obtained before surgery and at follow-up time points (3, 6, and 12 months where applicable). Statistical significance for repeated measures was assessed using the Friedman test, with intergroup comparisons performed using Dunn’s post hoc test with Bonferroni correction. BE, bariatric embolization; GP, gastric plication; SG, sleeve gastrectomy; BMI, body mass index; HbA1c, glycated hemoglobin.

Regarding weight-related outcomes, after 12 months, all groups showed significant weight loss (p < 0.001), the fastest in SG, the smallest in BE, and the difference between the groups remained at 6 and 12 months (p < 0.001) (**Figure 3B**). BMI decreased in all groups (p < 0.001), most rapidly in SG, more consistently in GP, after 12 months BMI in SG remained higher than in GP (p = 0.006) (**Figure 4A**).

In terms of glycemic control, improvements were observed across all groups: HbA1c decreased already after 3 months and significantly after 6 months (p < 0.001) (**Figure 4B**). Resistin decreased (p < 0.001), and adiponectin increased (p < 0.001) in all groups without intergroup differences (**Figures 4C, D**).

In terms of adipokine dynamics, ghrelin levels decreased in all groups (p < 0.001), with intergroup differences observed (**Figure 4E**). Leptin was significantly reduced in GP and SG (p < 0.001), and these differences persisted at 6 months (p = 0.028) (**Figure 4F**).

The distribution of rs1137101 and rs696217 genotypes among patients stratified by surgery type, which differed from that in the control group, is presented in **Supplementary Figure 1**. To further characterize these associations, a detailed analysis of weight-loss dynamics by genotype and intervention type was performed (**Supplementary Table 1**).

In line with the overall findings, the effect of the GHRL rs696217 polymorphism was procedure-specific. No significant associations with BMI or weight-loss parameters were observed after gastroplication (p > 0.05). In contrast, following sleeve gastrectomy, carriers of the T allele demonstrated significantly greater weight loss, including higher %TWL at 3, 6, and 12 months and greater %EWL at 12 months (all p < 0.001). A similar pattern was observed after bariatric embolization, where T allele carriers exhibited lower BMI at 12 months and higher %TWL and %EWL (p < 0.05).

The LEPR rs1137101 polymorphism was also associated with postoperative outcomes. The G allele was linked to higher %TWL at 12 months after gastroplication (p = 0.001), as well as increased %TWL and %EWL following sleeve gastrectomy (p = 0.006 and p = 0.016, respectively), supporting its role as a potential modifier of weight loss (**Supplementary Table 1**).

In addition, the GHRL T allele was associated with distinct hormonal changes depending on the intervention. After gastroplication, it was linked to higher baseline ghrelin (p = 0.042), followed by lower levels at 6 months (p < 0.001), and higher adiponectin at 3 months (p < 0.001). Following sleeve gastrectomy, T allele carriers exhibited higher baseline leptin (p = 0.040) and lower ghrelin at 3 and 6 months (p < 0.001). After embolization, the T allele was associated with lower HbA1c (p = 0.014), ghrelin (p = 0.0001 and p = 0.0004), and resistin (p = 0.0002).The LEPR G allele was additionally associated with higher adiponectin at 3 months after gastroplication (p = 0.030), as well as lower ghrelin at 6 months (p = 0.024) and higher baseline resistin (p = 0.030) following sleeve gastrectomy (**Supplementary Table 2**).

## 4 Discussion

The study demonstrated that increasing obesity severity is associated with significant alterations in the hormonal and metabolic profile: leptin and resistin increase, while adiponectin and total ghrelin decrease. These changes reflect progressive adipose tissue dysfunction and impaired metabolic homeostasis (45, 46). Our findings are consistent with previous studies demonstrating that leptin concentrations increase in proportion to adipose tissue mass, while adiponectin levels decrease with increasing body mass index (47–50). The imbalance of these hormones is considered an important mechanism for the development of metabolic disorders in obesity (51–53).

Elevated leptin levels in severe obesity may reflect the development of leptin resistance, in which increased hormone concentrations are not accompanied by effective regulation of appetite and energy balance (54–56). In contrast, adiponectin exerts an opposing metabolic role and shows a negative association with anthropometric indicators (57–59). Reduced adiponectin levels are associated with insulin resistance and deterioration of the metabolic profile (60, 61). Ghrelin is primarily produced in the stomach and, unlike classical adipokines, is not derived from adipose tissue and should therefore be considered separately from adipose tissue–derived hormones (62). The observed decrease in ghrelin levels in patients with higher obesity severity is consistent with other studies and may reflect adaptive changes in appetite regulation (63–65). Furthermore, ghrelin exists in different forms, including acylated and des-acyl ghrelin, which have distinct biological activities (66). The present study measured total ghrelin, which may not fully capture its complex physiological regulation, particularly after different bariatric procedures.

Genetic analysis showed that only the rs1137101 polymorphism in the leptin receptor gene, potentially affecting energy balance signaling pathways, was statistically significant (33, 67–69). The rs1137101 polymorphism (Q223R) of the LEPR gene has been widely investigated in studies of obesity and metabolic disorders (67). This variant may influence leptin receptor signaling and has been associated with alterations in appetite regulation, energy expenditure, and adiposity in different populations (55, 70). At the same time, the lack of associations with other genetic variants likely reflects the complex polygenic nature of obesity (71–74).

The identified associations of rs1137101 and rs696217 variants with obesity severity and treatment response may be explained by their functional roles on hormonal signaling pathways (75). The LEPR rs1137101 polymorphism (Q223R) has been linked to altered receptor activity and impaired leptin signaling, contributing to leptin resistance, increased appetite, and decreased energy expenditure (68).

Similarly, the GHRL rs696217 variant may modulate ghrelin secretion and its interaction with the growth hormone secretagogue receptor, affecting appetite regulation and metabolic adaptation after bariatric surgery (76). These mechanisms may partially explain the observed differences in weight loss dynamics and adipokine profiles among genotype carriers.

The present results are consistent with previous studies demonstrating that bariatric interventions result in significant weight loss and improvements in the metabolic profile during the first 6–12 months after surgery (77–80). It has been widely reported that weight loss after bariatric surgery is accompanied by pronounced changes in the adipokine profile, particularly a decrease in leptin levels and an increase in adiponectin concentrations (81–84). These changes reflect improved adipose tissue function, reduced systemic inflammation, and increased insulin sensitivity (85–88). The observed reductions in leptin and resistin levels, together with increased adiponectin, support the key role of adipokines in regulating metabolic homeostasis after weight loss (89, 90).

Emerging evidence also highlights the role of genetic factors in shaping individual responses to weight-loss interventions (91–94). In particular, polymorphisms in genes involved in appetite regulation, energy metabolism, and adipose tissue function may both susceptibility to obesity and treatment response (95–99). Gene variants involved in the leptin, adiponectin, or insulin signaling pathways are considered as potential modifiers of the metabolic response to bariatric surgery (100–104). In this context, genetic markers may serve as promising tools for personalized obesity treatment and predicting intervention outcomes (104–109).

Along with surgical methods, pharmacological approaches to treating obesity are actively evolving (110–112). Glucagon-like peptide-1 receptor agonists and dual incretin agonists have demonstrated significant efficacy in promoting weight loss and improve glycemic control in obese patients (113–116). However, the magnitude and durability of weight loss with pharmacotherapy are generally lower than after bariatric surgery, especially in severely obese patients (117–120). Metabolic drugs that improve tissue sensitivity to insulin and reduce hepatic glucose production may contribute to modest weight loss (121–124); in addition, some studies suggest they may affect adipokine regulation and metabolic signaling pathways (125–132). Diet therapy remains an important component of the comprehensive treatment of obesity, as rational eating patterns can improve the metabolic profile and reduce body weight (37, 133–135). It should also be noted that obesity is frequently associated with chronic diseases, such as metabolic syndrome and cardiovascular diseases (136–139), which can exacerbate metabolic disorders and affect the effectiveness of treatment (140–143).

The study has certain limitations: a relatively small sample size, an uneven distribution of patients by degree of obesity (in particular, a small number of cases at stage I), and a single-center design, which may limit the generalizability of the results. Further studies with larger samples are needed to more accurately assess the role of genetic factors in the development of obesity and response to bariatric treatment. An additional limitation is the inclusion of different types of bariatric procedures without fully stratified analysis, as these interventions have distinct physiological and metabolic effects. Baseline differences between surgical groups, particularly in obesity severity, may have influenced postoperative outcomes and should be considered when interpreting intergroup comparisons.

## 5 Conclusions

Changes in hormonal profiles and anthropometric parameters correlate with the degree of obesity: resistin increases, adiponectin and ghrelin decrease, while leptin increases. Genetic variants rs1137101 and rs696217 are associated with a predisposition to severe obesity, with reduced A-allele frequency and increased GG genotype in severe patients. Bariatric interventions effectively reduce BMI, improve glycemic control, and normalize hormones, with the type of surgery determining the speed and stability of weight loss. GHRL and LEPR polymorphisms modulate the response: the T-allele of rs696217 is associated with greater weight loss and hormonal changes, while the G-allele of rs1137101 affects adiponectin, resistin, and ghrelin levels. These results support the potential role of integrated hormonal and genetic profiling in personalizing obesity management; however, further large-scale studies are required to confirm these findings.

## Data Availability

The original contributions presented in the study are included in the article/**Supplementary Material**. Further inquiries can be directed to the corresponding authors.

## Glossary

BMI: Body mass index
HbA1c: Glycated hemoglobin
SNP: Single nucleotide polymorphism
LEPR: Leptin receptor gene
ADIPOQ: Adiponectin gene
GHRL: Ghrelin gene
BE: Bariatric embolization
GP: Gastric plication
SG: Sleeve gastrectomy
TWL: Total weight loss
ELISA: Enzyme-linked immunosorbent assay.
