# Supplementary figures and images for: Adipokine profiles and genetic variants of leptin receptor, adiponectin, and ghrelin pathways in obesity: prospective 12-month outcomes after bariatric interventions
Source: Front Endocrinol (Lausanne). 2026 May 20;17:1841033. doi: 10.3389/fendo.2026.1841033 (PMC13229757; doi:10.3389/fendo.2026.1841033)

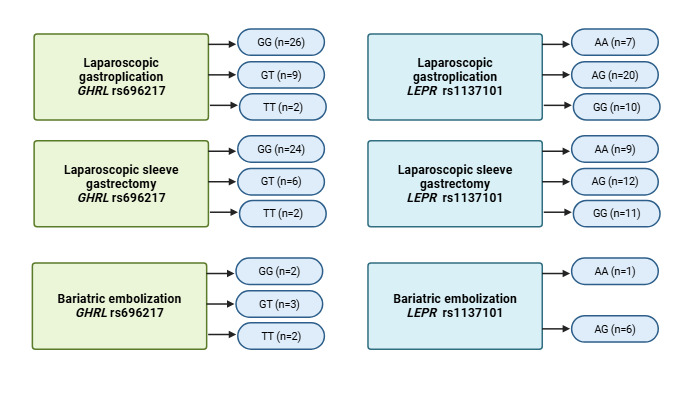

Supplement: Supplementary file 1 [file Image1.jpeg]
