# Supplementary material for: Adipokine profiles and genetic variants of leptin receptor, adiponectin, and ghrelin pathways in obesity: prospective 12-month outcomes after bariatric interventions
Source: Front Endocrinol (Lausanne). 2026 May 20;17:1841033. doi: 10.3389/fendo.2026.1841033 (PMC13229757; doi:10.3389/fendo.2026.1841033)
Supplement: Supplementary file 2 [file Table1.docx]

Figure 1 – Distribution of rs696217 and rs1137101 genotypes among patients undergoing different bariatric interventions: laparoscopic sleeve gastrectomy (n = 32), laparoscopic gastric plication (n = 37), and bariatric embolization (n = 7).

Table 1 – Association of *GHRL* (rs696217) and *LEPR* (rs1137101) polymorphisms with weight-loss outcomes after bariatric interventions

| Laparoscopic gastroplication | | | | | | |
| --- | --- | --- | --- | --- | --- | --- |
| Parameter | GHRL C214A (rs696217) | | | | р | |
|  | Allele G | | Allele T | |  |  |
| 1 | 2 | | 3 | | 4 | |
| BMI (before surgery, kg/m²) | 40.39±3.23 | | 38.83±2.98 | | **0.164** | |
| BMI (3 months post-op, kg/m²) | 36.13±3.76 | | 34.73±2.24 | | **0.210** | |
| BMI (6 months post-op, kg/m²) | 33.90±3.65 | | 32.81±3.09 | | **0.327** | |
| BMI (12 months post-op, kg/m²) | 32.33±4.32 | | 31.27±2.76 | | **0.341** | |
| TWL (3 months post-op, %) | 11.10% | | 10.14% | | **0.388** | |
| TWL (6 months post-op, %) | 16.52% | | 14.97% | | **0.167** | |
| TWL (12 months post-op, %) | 20.18% | | 18.77% | | **0.207** | |
| EWL (12 months post-op, %) | 43.97% | | 42.29% | | **0.135** | |
| Laparoscopic sleeve gastrectomy | | | | | | |
| Parameter | | GHRL C214A (rs696217) | | | | р |
|  |  | Allele G | | Allele T | |  |
| BMI (before surgery, kg/m²) | | 45.13±3.43 | | 51.19±4.32 | | <0.001* |
| BMI (3 months post-op, kg/m²) | | 39.81±3.04 | | 43.47±3.67 | | **0.002** |
| BMI (6 months post-op, kg/m²) | | 36.38±4.01 | | 36.84±3.45 | | **0.678** |
| BMI (12 months post-op, kg/m²) | | 32.63±3.98 | | 30.30±3.32 | | **0.042*** |
| TWL (3 months post-op, %) | | 11.48% | | 16.66% | | <0.001* |
| TWL (6 months post-op, %) | | 18.73% | | 29.16% | | <0.001* |
| TWL (12 months post-op, %) | | 27.09% | | 41.35% | | <0.001* |
| EWL (12 months post-op, %) | | 52.98% | | 72.57% | | <0.001* |
| Bariatric gastric artery embolization | | | | | | |
| Parameter | GHRL C214A (rs696217) | | | | р | |
|  | Allele G | | | Allele T |  |  |
| BMI (before surgery, kg/m²) | 38.77±3.42 | | | 37.62±3.11 | **0.354** | |
| BMI (3 months post-op, kg/m²) | 37.31±2.98 | | | 35.24±3.87 | **0.132** | |
| BMI (6 months post-op, kg/m²) | 35.55±3.17 | | | 33.48±3.98 | **0.132** | |
| BMI (12 months post-op, kg/m²) | 34.77±3.67 | | | 31.16±2.87 | **0.030*** | |
| TWL (3 months post-op, %) | 3.05% | | | 6.28% | **0.047*** | |
| TWL (6 months post-op, %) | 7.61% | | | 10.92% | **0.039*** | |
| TWL (12 months post-op, %) | 9.64% | | | 14.43% | **0.012*** | |
| EWL (12 months post-op, %) | 22.96% | | | 34.99% | **0.0003*** | |
| Laparoscopic gastroplication | | | | | | |
| Parameter | LEPR Q223R (rs1137101) | | | | р | |
|  | Allele A | | | Allele G |  |  |
| BMI (before surgery, kg/m²) | 38.26±4.50 | | | 40.31±4.03 | **0.070** | |
| BMI (3 months post-op, kg/m²) | 34.91±4.33 | | | 35.90±3.98 | **0.373** | |
| BMI (6 months post-op, kg/m²) | 33.47±4.03 | | | 33.72±4.27 | **0.821** | |
| BMI (12 months post-op, kg/m²) | 32.09±3.97 | | | 32.00±4.06 | **0.935** | |
| TWL (3 months post-op, %) | 9.46% | | | 11.13% | **0.137** | |
| TWL (6 months post-op, %) | 14.46% | | | 16.43% | **0.081** | |
| TWL (12 months post-op, %) | 16.66% | | | 20.48% | **0.001** | |
| EWL (12 months post-op, %) | 37.60% | | | 44.84% | **0.098** | |
| Laparoscopic sleeve gastrectomy | | | | | | |
| Parameter | LEPR Q223R (rs1137101) | | | | р | |
|  | Allele A | | | Allele G |  |  |
| BMI (before surgery, kg/m²) | 42.09±4.77 | | | 48.43±4.77 | **0.009** | |
| BMI (3 months post-op, kg/m²) | 37.15±5.02 | | | 42.13±4.31 | **0.038** | |
| BMI (6 months post-op, kg/m²) | 33.86±4.49 | | | 37.52±4.67 | **0.122** | |
| BMI (12 months post-op, kg/m²) | 30.81±3.89 | | | 32.53±4.32 | **0.460** | |
| TWL (3 months post-op, %) | 11.20% | | | 13.34% | **0.359** | |
| TWL (6 months post-op, %) | 18.54% | | | 22.43% | **0.101** | |
| TWL (12 months post-op, %) | 25.81% | | | 32.55% | **0.006*** | |
| EWL (12 months post-op, %) | 53.66% | | | 59.53% | **0.016*** | |
| * – statistically significant difference (p < 0.05). | | | | | | |

Table 2 – Association of *GHRL* (rs696217) and *LEPR* (rs1137101) polymorphisms with metabolic and adipokine profiles after bariatric interventions

| Laparoscopic gastroplication | | | | | | |  |
| --- | --- | --- | --- | --- | --- | --- | --- |
| Parameter | GHRL C214A (rs696217) | | | | р | |  |
|  | Allele G | | Allele Т | |  |  |  |
| 1 | 2 | | 3 | | 4 | |  |
| HbA1c, % (before surgery) | 6.11±0.11 | | 6.04±0.12 | | 0.715 | |  |
| HbA1c, % (3 months) | 5.44±0.09 | | 5.35±0.13 | | 0.639 | |  |
| HbA1c, % (6 months) | 4.96±0.12 | | 4.85±0.10 | | 0.566 | |  |
| Leptin, ng/mL (before surgery) | 43.75±4.67 | | 54.66±4.87 | | 0.108 | |  |
| Leptin, ng/mL (3 months) | 38.80±5.11 | | 32.48±4.12 | | 0.340 | |  |
| Leptin, ng/mL (6 months) | 30.10±4.65 | | 24.63±4.06 | | 0.352 | |  |
| Total ghrelin, ng/mL (before surgery) | 738.94±9.12 | | | 767.11±9.32 | | 0.042* |  |
| Total ghrelin, ng/mL (3 months) | 435.38±8.43 | | | 453.38±8.56 | | 0.100 |  |
| Total ghrelin, ng/mL (6 months) | 352.93±10.43 | | | 281.25±9.02 | | <0.001* |  |
| Adiponectin, µg/mL (before surgery) | 6.34±0.54 | | | 6.14±0.83 | | 0.298 |  |
| Adiponectin, µg/mL (3 months) | 8.42±0.87 | | | 10.03±0.33 | | <0.001* |  |
| Adiponectin, µg/mL (6 months) | 10.07±0.45 | | | 10.92±0.87 | | 0.338 |  |
| Resistin, ng/mL (before surgery) | 8.43±0.76 | | | 8.32±0.23 | | 0.915 |  |
| Resistin, ng/mL (3 months) | 7.49±0.43 | | | 7.24±0.92 | | 0.809 |  |
| Resistin, ng/mL (6 months) | 6.67±0.32 | | | 6.56±0.12 | | 0.915 |  |
| Laparoscopic sleeve gastrectomy | | | | | | | |
| Parameter | GHRL C214A (rs696217) | | | | | р | |
|  | Allele G | | | Allele Т | |  |  |
| HbA1c, % (before surgery) | 6.01±0.12 | | | 6.43±0.09 | | **0.687** | |
| HbA1c, % (3 months) | 5.69±0.08 | | | 5.75±0.10 | | **0.954** | |
| HbA1c, % (6 months) | 5.37±0.11 | | | 4.88±0.13 | | **0.639** | |
| Leptin, ng/mL (before surgery) | 44.89±4.32 | | | 62.61±4.99 | | **0.040*** | |
| Leptin, ng/mL (3 months) | 28.57±3.99 | | | 25.78±4.05 | | **0.599** | |
| Leptin, ng/mL (6 months) | 27.07±4.65 | | | 21.93±4.76 | | **0.336** | |
| Total ghrelin, ng/mL (before surgery) | 602.28±7.65 | | | 618.91±8.45 | | **0.213** | |
| Total ghrelin, ng/mL (3 months) | 384.21±8.02 | | | 266.19±8.92 | | <0.001* | |
| Total ghrelin, ng/mL (6 months) | 246.08±7.54 | | | 131.16±7.05 | | <0.001* | |
| Adiponectin, µg/mL (before surgery) | 6.09±0.43 | | | 5.98±0.76 | | **0.920** | |
| Adiponectin, µg/mL (3 months) | 8.46±0.54 | | | 10.61±0.43 | | **0.059** | |
| Adiponectin, µg/mL (6 months) | 10.30±0.78 | | | 11.64±0.53 | | **0.232** | |
| Resistin, ng/mL (before surgery) | 9.12±0.35 | | | 8.96±0.22 | | **0.885** | |
| Resistin, ng/mL (3 months) | 7.93±0.54 | | | 8.01±0.78 | | **0.942** | |
| Resistin, ng/mL (6 months) | 7.14±0.87 | | | 7.17±0.21 | | **0.978** | |
| Bariatric gastric artery embolization | | | | | | | |
| Parameter | | GHRL C214A (rs696217) | | | | р | |
|  |  | Allele G | | Allele Т | |  |  |
| HbA1c, % (before surgery) | | 6.05±0.09 | | 5.96±0.12 | | **0.938** | |
| HbA1c, % (3 months) | | 5.72±0.14 | | 5.62±0.11 | | **0.931** | |
| HbA1c, % (6 months) | | 5.36±0.08 | | 4.65±0.09 | | **0.014*** | |
| Leptin, ng/mL (before surgery) | | 35.36±3.98 | | 39.11±4.21 | | **0.569** | |
| Leptin, ng/mL (3 months) | | 29.17±4.01 | | 25.54±4.11 | | **0.581** | |
| Leptin, ng/mL (6 months) | | 26.94±3.67 | | 23.03±4.12 | | **0.554** | |
| Total ghrelin, ng/mL (before surgery) | | 822.23±8.32 | | 839.66±9.01 | | **0.210** | |
| Total ghrelin, ng/mL (3 months) | | 430.06±7.99 | | 267.87±8.65 | | **0.0001*** | |
| Total ghrelin, ng/mL (6 months) | | 289.66±8.23 | | 164.11±8.34 | | **0.0004*** | |
| Adiponectin, µg/mL (before surgery) | | 6.15±0.54 | | 6.66±0.42 | | **0.666** | |
| Adiponectin, µg/mL (3 months) | | 9.50±0.67 | | 10.45±0.58 | | **0.435** | |
| Adiponectin, µg/mL (6 months) | | 10.24±0.77 | | 11.10±0.43 | | **0.477** | |
| Resistin, ng/mL (before surgery) | | 8.81±0.54 | | 8.18±0.21 | | **0.986** | |
| Resistin, ng/mL (3 months) | | 8.53±0.72 | | 7.73±0.56 | | **0.945** | |
| Resistin, ng/mL (6 months) | | 8.33±0.22 | | 6.21±0.71 | | 0.0002***** | |
| Laparoscopic gastroplication | | | | | | | |
| Parameter | LEPR Q223R (rs1137101) | | | | | р | |
|  | Allele G | | | Allele Т | |  |  |
| HbA1c, % (before surgery) | 6.29±0.11 | | | 6.04±0.15 | | **0.608** | |
| HbA1c, % (3 months) | 5.77±0.08 | | | 5.33±0.13 | | **0.369** | |
| HbA1c, % (6 months) | 5.21±0.09 | | | 4.86±0.13 | | **0.474** | |
| Leptin, ng/mL (before surgery) | 37.50±4.78 | | | 43.43±5.67 | | **0.227** | |
| Leptin, ng/mL (3 months) | 34.01±5.32 | | | 37.60±6.02 | | **0.461** | |
| Leptin, ng/mL (6 months) | 26.57±4.22 | | | 28.92±4.78 | | **0.629** | |
| Total ghrelin, ng/mL (before surgery) | 814.64±9.32 | | | 731.60±9.65 | | **0.529** | |
| Total ghrelin, ng/mL (3 months) | 461.85±8.67 | | | 435.80±10.34 | | **0.069** | |
| Total ghrelin, ng/mL (6 months) | 339.86±10.32 | | | 329.70±9.45 | | **0.469** | |
| Adiponectin, µg/mL (before surgery) | 6.73±0.32 | | | 6.17±0.67 | | **0.255** | |
| Adiponectin, µg/mL (3 months) | 8.03±0.46 | | | 9.12±0.54 | | **0.030** | |
| Adiponectin, µg/mL (6 months) | 9.79±0.33 | | | 10.45±0.32 | | **0.181** | |
| Resistin, ng/mL (before surgery) | 8.53±0.83 | | | 8.37±0.67 | | **0.742** | |
| Resistin, ng/mL (3 months) | 7.51±0.91 | | | 7.40±0.87 | | **0.821** | |
| Resistin, ng/mL (6 months) | 6.63±0.21 | | | 6.64±0.34 | | **0.983** | |
| Laparoscopic sleeve gastrectomy | | | | | | | |
| Parameter | | LEPR Q223R (rs1137101) | | | | р | |
|  |  | Allele A | | Allele G | |  |  |
| HbA1c, % (before surgery) | | 5.93±0.13 | | 6.18±0.11 | | **0.608** | |
| HbA1c, % (3 months) | | 5.61±0.16 | | 5.74±0.09 | | **0.789** | |
| HbA1c, % (6 months) | | 5.27±0.12 | | 5.37±0.14 | | **0.837** | |
| Leptin, ng/mL (before surgery) | | 45.27±5.45 | | 54.82±5.78 | | **0.133** | |
| Leptin, ng/mL (3 months) | | 29.17±4.87 | | 27.37±4.89 | | **0.773** | |
| Leptin, ng/mL (6 months) | | 27.99±4.22 | | 24.92±5.02 | | **0.623** | |
| Total ghrelin, ng/mL (before surgery) | | 786.31±10.34 | | 656.05±10.05 | | **0.134** | |
| Total ghrelin, ng/mL (3 months) | | 373.62±9.34 | | 345.43±11.45 | | **0.119** | |
| Total ghrelin, ng/mL (6 months) | | 247.34±10.32 | | 205.61±9.43 | | **0.024** | |
| Adiponectin, µg/mL (before surgery) | | 6.34±0.54 | | 5.95±0.76 | | **0.425** | |
| Adiponectin, µg/mL (3 months) | | 8.49±0.78 | | 9.20±0.86 | | **0.151** | |
| Adiponectin, µg/mL (6 months) | | 10.68±0.75 | | 10.62±0.35 | | **0.902** | |
| Resistin, ng/mL (before surgery) | | 8.28±0.35 | | 9.37±0.78 | | **0.030** | |
| Resistin, ng/mL (3 months) | | 7.31±0.67 | | 8.21±0.86 | | **0.071** | |
| Resistin, ng/mL (6 months) | | 6.72±0.32 | | 7.26±0.24 | | **0.272** | |
| * – statistically significant difference (p < 0.05). | | | | | | | |
